# Impact of anesthetic agents on overall and recurrence-free survival in patients undergoing esophageal cancer surgery: A retrospective observational study

**DOI:** 10.1038/s41598-017-14147-9

**Published:** 2017-10-25

**Authors:** In-Jung Jun, Jun-Young Jo, Jong-Il Kim, Ji-Hyun Chin, Wook-Jong Kim, Hyeong Ryul Kim, Eun-Ho Lee, In-Cheol Choi

**Affiliations:** 1 Department of Anesthesiology and Pain Medicine, Asan Medical Center, University of Ulsan College of Medicine, Seoul, Korea; 2 Department of Thoracic and Cardiovascular Surgery, Asan Medical Center, University of Ulsan College of Medicine, Seoul, Korea

## Abstract

Given that surgical stress response and surgical excision may increase the likelihood of post-surgery cancer dissemination and metastasis, the appropriate choice of surgical anesthetics may be important for oncologic outcomes. We evaluated the association of anesthetics used for general anesthesia with overall survival and recurrence-free survival in patients who underwent esophageal cancer surgery. Adult patients (922) underwent elective esophageal cancer surgery were included. The patients were divided into two groups according to the anesthetics administered during surgery: volatile anesthesia (VA) or intravenous anesthesia with propofol (TIVA). Propensity score and Cox regression analyses were performed. There were 191 patients in the VA group and 731 in the TIVA group. In the entire cohort, VA was independently associated with worse overall survival (HR 1.58; 95% CI 1.24–2.01; *P* < 0.001) and recurrence-free survival (HR 1.42; 95% CI 1.12–1.79; *P* = 0.003) after multivariable analysis adjustment. Similarly, in the propensity score matched cohorts, VA was associated with worse overall survival (HR 1.45; 95% CI 1.11–1.89; *P* = 0.006) and recurrence-free survival (HR 1.44; 95% CI 1.11–1.87; *P* = 0.006). TIVA during esophageal cancer surgery was associated with better postoperative survival rates compared with volatile anesthesia.

## Introduction

Esophageal cancer is one of the most fatal malignancies with very poor overall 5-year survival rates (10–40%)^1,2^. Surgical removal is still considered the mainstay therapy for longer survival rates in esophageal cancer. However, since correlation between surgery and postoperative metastasis was suggested^3^, the importance of the perioperative period in cancer metastasis has been reiterated^4,5^. Indeed, surgery itself may be a risk factor of loco-regional recurrence and distant metastasis through destruction of tumor cells and vasculature as well as its effects on neuroendocrine factors^6,7^. Thus, given the contribution of recurrence to poor prognosis after esophageal cancer surgery^8,9^, preserving the host immune function against micrometastasis of remnant cancer cells during surgery may be important for improving postoperative oncologic outcomes^5^.

Patients undergoing esophageal surgery for cancer require general anesthesia with either a volatile (isoflurane, sevoflurane, and desflurane) or an intravenous anesthetic such as propofol. To date, there is no definitive evidence supporting superiority of the use of either a volatile or an intravenous anesthetic. However, several studies have reported that anesthetic techniques used during cancer surgery may affect cancer recurrence and ultimately survival^5,10–13^. While volatile anesthetics may exert immunosuppressive effects, propofol may rather have protective effects through the enhancement of antitumor immunity^5,10,14^. Thus, intravenous anesthesia with propofol may be preferable to volatile anesthetics during cancer surgery. Furthermore, it has been suggested that volatile anesthetics during cancer surgery may be associated with poor oncologic outcomes^11–13^. Therefore, we performed a retrospective study to assess the relationship of anesthesia with overall and recurrence-free survival rates in patients following esophageal cancer surgery. We hypothesized that patients who received intravenous anesthesia with propofol might have better overall and recurrence-free survivals after esophageal cancer surgery compared with patients who have received a volatile anesthetic.

## Results

Of the 1,084 patients who underwent esophagectomy within the study period, 162 met exclusion criteria and a total of 922 patients remained for analysis (volatile anesthesia [VA]: 191 and intravenous anesthesia with propofol [TIVA]: 731, Fig. 1). In the VA group, patients received either isoflurane (n = 53), sevoflurane (n = 23), or desflurane (n = 115) for induction and maintenance of anesthesia. The baseline and perioperative characteristics of the study groups in the entire cohort are illustrated in Table 1. Compared with TIVA, patients in the VA group were more likely to have histories of chronic obstructive pulmonary disease, lower preoperative total bilirubin and albumin concentrations, and diuretic therapy. Additionally, the use of epidural analgesia for postoperative pain control and postoperative body weight gain (%) were lower in the VA group compared with the TIVA group.

**Figure 1:**
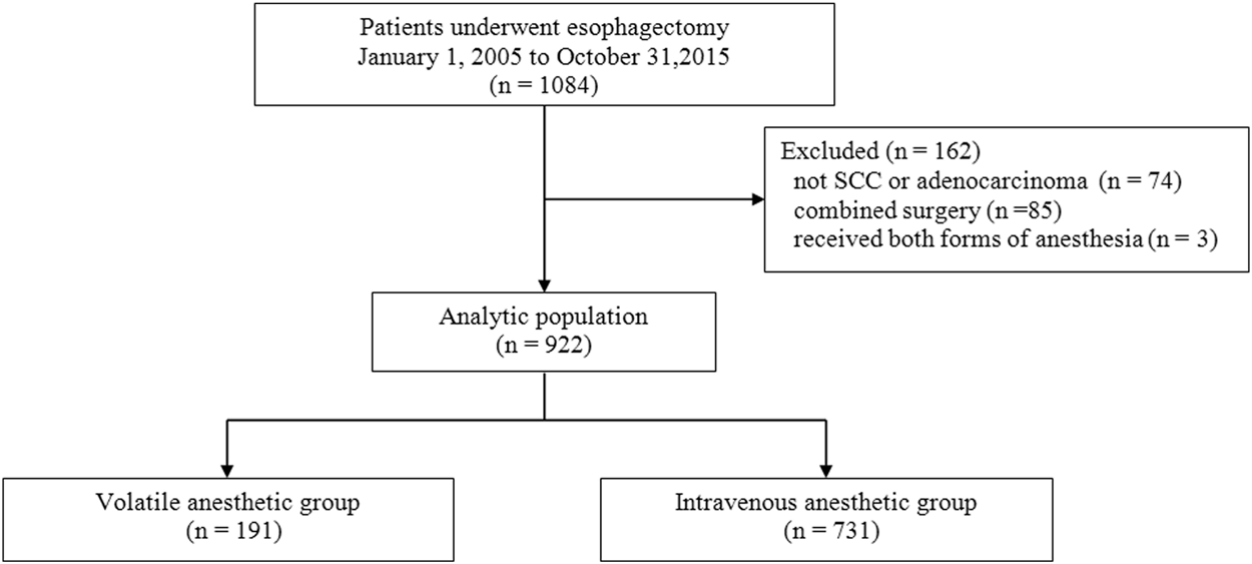
Study flow diagram. SCC = squamous cell carcinoma.

**Table 1:** Baseline and perioperative characteristics

Variable	VA group	TIVA group	*P* value	STD
N	191	731		
Demographics				
Age (yr)	63.1 ± 7.2	62.5 ± 7.9	0.344	0.079
Male gender (n, %)	183 (95.8)	683 (93.4)	0.221	0.106
Clinical characteristics				
Body mass index (kg/m^2^)	23.0 ± 3.1	23.1 ± 2.8	0.558	0.047
ASA class (n, %)			0.962	0.022
I	15 (7.9)	60 (8.2)		
II	171 (89.5)	654 (89.5)		
III	5 (2.6)	17 (2.3)		
Medical history (n, %)				
Diabetes mellitus	33 (17.3)	120 (16.4)	0.776	0.023
Hypertension	74 (38.7)	273 (37.4)	0.723	0.029
Dyslipidemia	21 (11.0)	52 (7.1)	0.077	0.136
Coronary artery disease	6 (3.1)	11 (1.5)	0.137	0.109
Cerebral vascular disease	7 (3.7)	29 (4.0)	0.848	0.016
Peripheral vascular disease	3 (1.6)	21 (2.9)	0.445	0.088
COPD	9 (4.7)	13 (1.8)	0.029	0.166
eGFR < 60 mL/min/1.73 m^2^	10 (5.2)	28 (3.8)	0.384	0.068
Liver disease	14 (7.3)	83 (11.4)	0.107	0.139
Smoker, current	49 (25.7)	182 (24.9)	0.830	0.017
Alcohol status	169 (88.5)	641 (87.7)	0.765	0.025
Chemo-radiation therapy	79 (41.4)	299 (40.9)	0.909	0.009
Laboratory data				
Hematocrit (%)	37.4 ± 5.2	38.0 ± 4.8	0.099	0.131
Creatinine (mg/dL)	0.9 [0.8–1.0]	0.8 [0.7–0.9]	0.262	0.090
Total bilirubin (mg/dL)	0.5 [0.4–0.7]	0.6 [0.4–0.8]	0.008	0.210
Albumin (g/dL)	3.6 ± 0.4	3.7 ± 0.4	0.022	0.184
LVEF (%)	61.9 ± 4.3	61.8 ± 4.6	0.803	0.023
FVC (% predicted)	94 [86–103]	92 [84–100]	0.041	0.125
FEV_1_ (% predicted)	93 [82–103]	92 [83–101]	0.964	0.020
FEV_1_ / FVC	71.5 ± 10.6	73.1 ± 9.0	0.052	0.170
Medication (n, %)				
ACEI or ARB	33 (17.3)	140 (19.2)	0.555	0.049
Beta blocker	18 (9.4)	49 (6.7)	0.197	0.100
Calcium channel blocker	36 (18.9)	145 (19.8)	0.760	0.025
Insulin	32 (16.8)	112 (15.3)	0.627	0.039
Oral hypoglycemic agent	26 (13.6)	77 (10.5)	0.229	0.095
Statin	15 (7.9)	70 (9.6)	0.464	0.061
Aspirin	18 (9.4)	41 (5.6)	0.055	0.145
Plavix	6 (3.1)	11 (1.5)	0.137	0.109
Diuretics	12 (6.3)	84 (11.5)	0.036	0.184
Intraoperative data				
Anesthesia time (min)	426.9 ± 91.9	418.4 ± 104.9	0.271	0.086
**One lung ventilation time (min)**	**125.6 ± 51.9**	**120.7 ± 58.3**	**0.116**	**0.090**
Crystalloid (L)	1.6 [1.2–2.2]	1.7 [1.1–2.4]	0.246	0.108
Colloid (L)	0.9 [0–1.0]	0.9 [0.5–1.0]	0.106	0.152
Packed red blood cell (unit)	0 [0–0]	0 [0–0]	0.822	0.014
Use of packed red blood cell	23 (12.0)	84 (11.5)	0.932	0.017
Fresh frozen plasma (unit)	0 [0–0]	0 [0–0]	0.462	0.061
**Minimally invasive surgery**	**38 (19.9)**	**128 (17.5)**	**0.445**	**0.061**
Squamous cell carcinoma	185 (96.9)	716 (97.9)	0.412	0.069
**McKeown operation**	**71 (37.2)**	**265 (36.3)**	**0.880**	**0.019**
Pathologic stage of cancer			0.361	0.163
0	44 (23.0)	167 (22.8)		
I	72 (37.7)	298 (40.8)		
II	39 (20.4)	170 (23.3)		
III	33 (17.3)	88 (12.0)		
IV	3 (1.6)	8 (1.1)		
Postoperative data				
Epidural analgesia	146 (76.4)	659 (90.2)	<0.001	0.374
Weight gain (%)	0.4 [−1.1–1.7]	0.9 [−0.4–2.4]	0.001	0.194
Chemo-radiation therapy	30 (15.7)	108 (14.8)	0.748	0.026

Data are expressed as number of patients (%), mean ± standard deviation, or median [first-third quartiles].

VA = volatile inhalational anesthesia; TIVA = total intravenous anesthesia; STD = standardized difference; ASA = American Society of Anesthesiology; COPD = chronic obstructive pulmonary disease; eGFR = estimated glomerular filtration rate; LVEF = left ventricle ejection fraction; FVC = forced vital capacity; FEV_1_ = forced expiratory volume in 1 second; ACEI = angiotensin-converting enzyme inhibitor; ARB = angiotensin receptor blocker.

Postoperative short-term outcomes are shown in Table 2. The incidence of postoperative myocardial dysfunction and sepsis was lower in the VA group compared with the TIVA group, but postoperative intensive care unit and hospital stay together with the incidence of other complications were comparable between the two groups.

**Table 2:** Postoperative outcomes

Outcome	VA group	TIVA group	*P* value
N	191	731	
Complications			
Cardio-cerebrovascular			
Myocardial infarction	3 (1.6)	2 (0.3)	0.063
Ventricular arrhythmia	1 (0.5)	3 (0.4)	1.000
Myocardial dysfunction	16 (8.4)	135 (18.5)	0.001
Stroke	2 (1.0)	4 (0.5)	0.610
Respiratory			
Mechanical ventilation > 48 h	10 (5.2)	47 (6.4)	0.659
Pneumonia	30 (15.7)	110 (15.0)	0.910
ALI or ARDS	5 (2.6)	23 (3.1)	0.817
Renal			
Acute kidney injury	48 (25.1)	206 (28.2)	0.454
Renal replacement therapy	3 (1.6)	11 (1.5)	1.000
Sepsis	12 (6.3)	87 (11.9)	0.036
Multi-organ failure	4 (2.1)	15 (2.1)	1.000
Intensive care unit stay (h)	25 [21–46]	24 [21–45]	0.150
Hospital stay (d)	14 [12–19]	13 [11–19]	0.157
In-hospital death	6 (3.1)	17 (2.3)	0.702

Data are expressed as number of patients (%) or median [first-third quartiles].

VA = volatile inhalational anesthesia; TIVA = total intravenous anesthesia; ALI = acute lung injury; ARDS = acute respiratory distress syndrome.

Median follow-up for all patients was 37.9 months (interquartile range: 17.6–67.5 months), 25.9 months (interquartile range: 14.2–47.8 months) for VA group, and 41.4 months (interquartile range: 18.4–70.0 months) for TIVA group.

**Figure 2:**
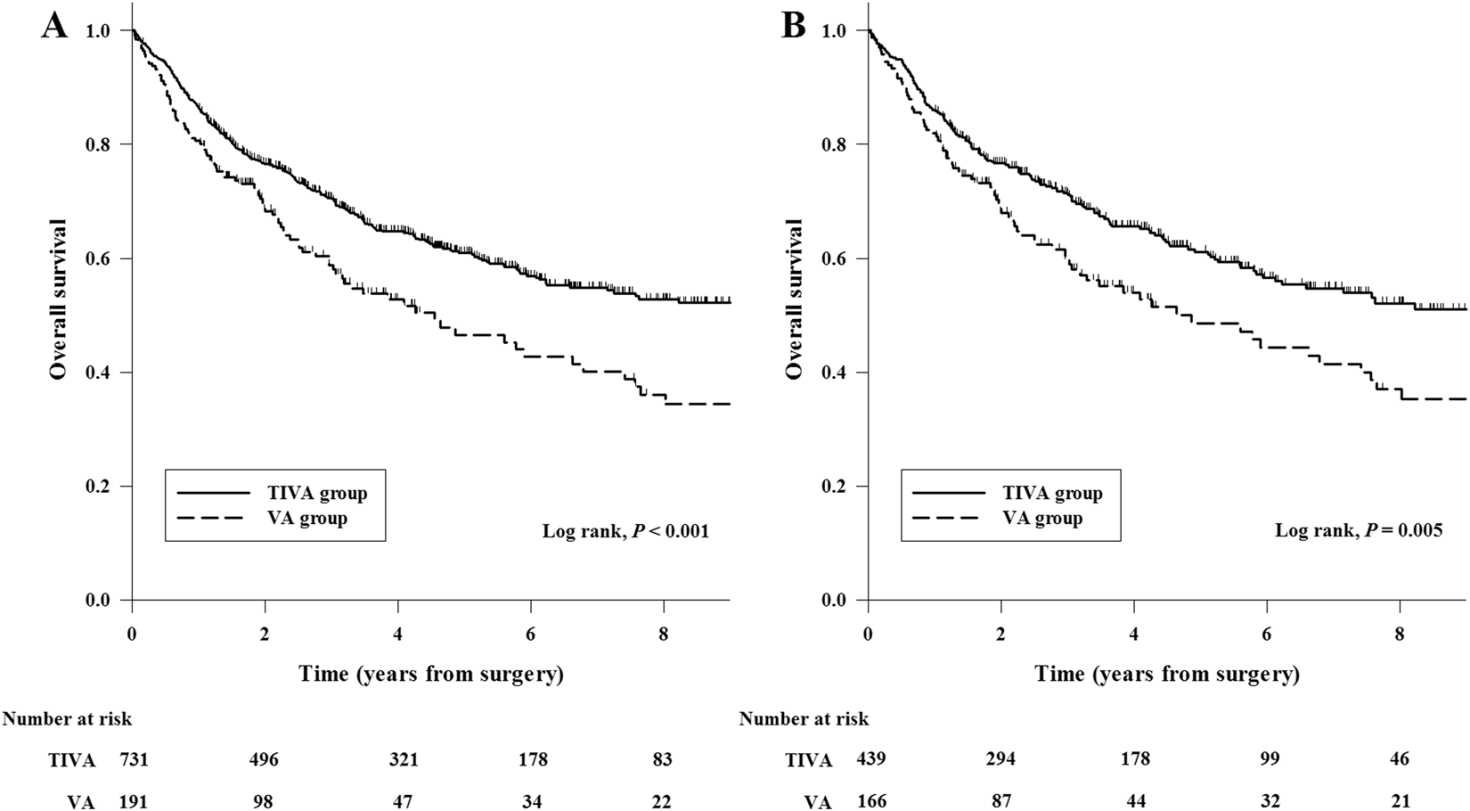
Kaplan-Meier overall survival curves from the date of esophageal cancer surgery by anesthesia type in the total cohort (**A**) and the propensity matched cohort (**B**).

**Figure 3:**
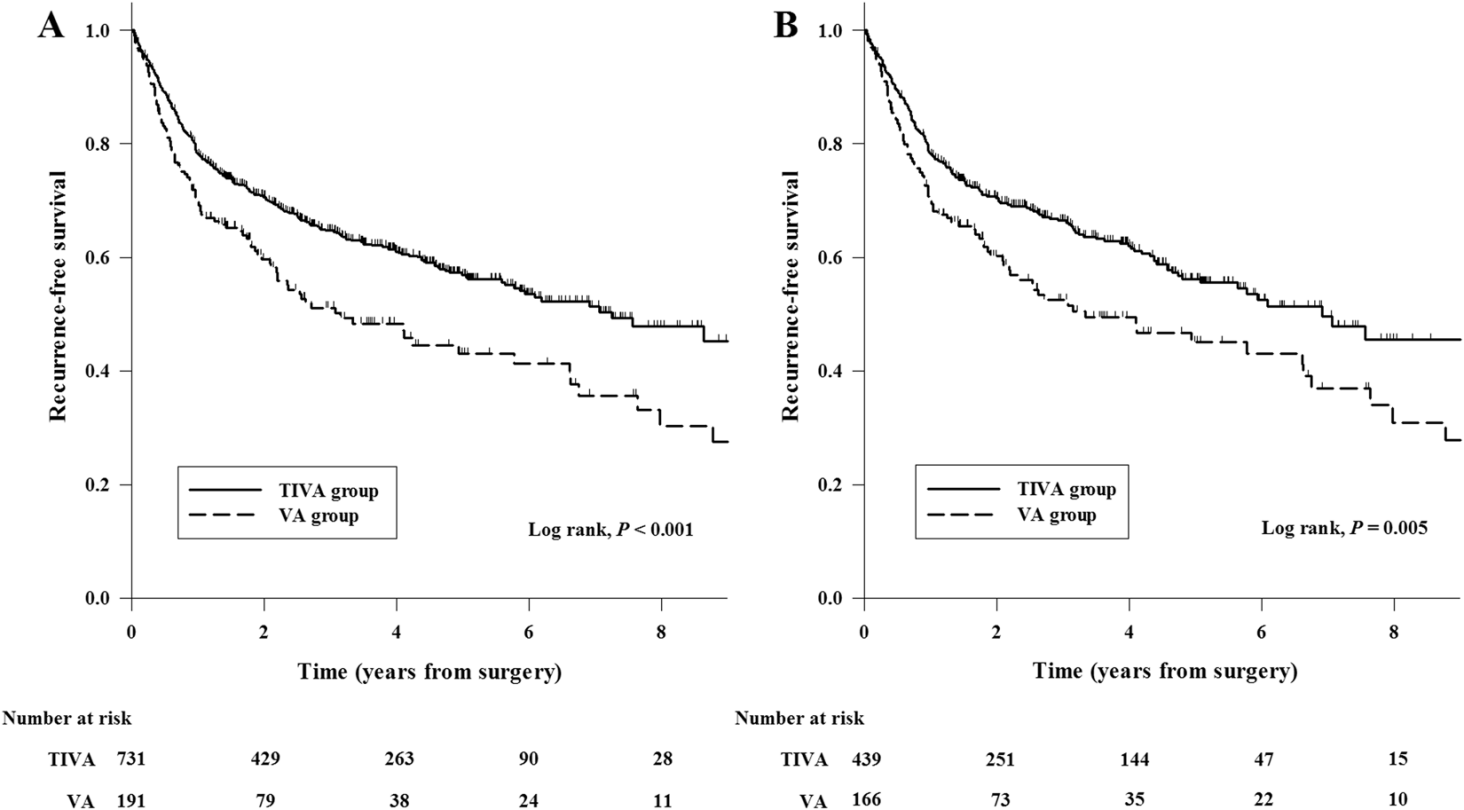
Kaplan-Meier recurrence-free survival curves from the date of esophageal cancer surgery by anesthesia type in the total cohort (**A**) and the propensity matched cohort (**B**).

In the entire cohort, the overall mortality rate during follow-up was 41.4% (382/922) in all patients, 51.3% (98/191) in the VA group, and 36.4% (284/731) in the TIVA group. The Kaplan-Meier curve showed that 1-, 3-, and 5-year overall survival rates for VA group were 80.1% (95% CI, 74.4–85.8), 58.0% (95% CI, 50.4–65.6), and 46.6% (95% CI, 37.8–55.4), respectively. On the other hand, the 1-, 3-, and 5-year overall survival rates for TIVA group were 86.4% (95% CI, 83.9–88.9), 70.4% (95% CI, 67.0–73.8), and 61.2% (95% CI, 57.3–65.1), respectively. These data suggest a significantly lower overall survival rate in the VA group compared with the TIVA group (*P* < 0.001, Fig. 2A). The recurrence-free survival rates in the VA group were also lower than those in the TIVA group (69.2% [95% CI, 62.6–75.8] *vs*. 78.2% [95% CI, 75.2–81.2] in 1-year, 50.2% [95% CI, 42.3–58.1] *vs*. 64.5% [95% CI, 60.9–68.2] in 3-year, and 43.0% [95% CI, 34.4–51.6] *vs*. 56.9% [95% CI, 52.8–61.0] in 5-year; *P* < 0.001, Fig. 3A). In univariate analysis, volatile anesthesia was associated with both worse overall survival (HR 1.50; 95% CI 1.19–1.89; *P* = 0.001) and recurrence-free survival (HR 1.48; 95% CI 1.18–1.86; *P* = 0.001). After adjusting by multivariable, Cox regression analysis demonstrated that volatile anesthesia was independently associated with both worse overall survival (HR 1.58; 95% CI 1.24–2.01; *P* < 0.001) and recurrence-free survival (HR 1.42; 95% CI 1.12–1.79; *P* = 0.003) (Table 3). Other variables associated with the overall survival are shown in Supplementary Table 2.

**Table 3:** Impact of anesthetic type on overall survival and RF survival

Outcome	Model	Hazard Ratio (95% CI)	*P* value
Overall survival	Unadjusted	1.50 (1.19–1.89)	0.001
	Multivariable adjusted*	1.58 (1.24–2.01)	<0.001
	PS matching†	1.45 (1.11–1.89)	0.006
	PS matching and adjusted by covariate‡	1.57 (1.19–2.07)	0.001
RF survival	Unadjusted	1.48 (1.18–1.86)	0.001
	Multivariable adjusted§	1.42 (1.12–1.79)	0.003
	PS matching†	1.44 (1.11–1.87)	0.006
	PS matching and adjusted by covariate‖	1.36 (1.04–1.77)	0.024

Hazard ratios are for the VA group relative to the TIVA group.

*Adjusted by age, body mass index, diabetes mellitus, preoperative chemo-radiation therapy, preoperative albumin levels, pathologic stage of cancer, pRBC given intraoperatively, and postoperative complications (myocardial dysfunction and sepsis).

†Cox proportional hazards model was applied by using propensity score-based matching with robust standard errors.

‡Adjusted by pathologic stage of cancer, pRBC given intraoperatively, and postoperative complications (myocardial dysfunction and sepsis).

§Adjusted by age, body mass index, preoperative chemo-radiation therapy, preoperative albumin levels, pathologic stage of cancer, and pRBC given intraoperatively.

‖Adjusted by pathologic stage of cancer, pRBC given intraoperatively, and postoperative chemo-radiation therapy.

RF = Recurrence-free; CI = confidence interval; PS = propensity score; VA = volatile inhalational anesthesia; TIVA = total intravenous anesthesia; pRBC = packed red blood cell.

In the propensity score matched cohorts, both survival rates were lower in the VA group compared with the TIVA group: overall survival rate (81.3% [95% CI, 75.4–87.2] *vs*. 85.8% [95% CI, 82.5–89.1] in 1-year, 59.8% [95% CI, 51.8–67.8] *vs*. 71.2% [95% CI, 66.8–75.6] in 3-year, and 48.6% [95% CI, 39.5–57.8] *vs*. 61.2% [95% CI, 56.1–66.3] in 5-year; *P* = 0.005, Fig. 2B); recurrence-free survival rates (70.1% [95% CI, 63.1–77.1] *vs*. 78.3% [95% CI, 74.4–82.2] in 1-year, 52.5% [95% CI, 44.3–60.7] *vs*. 66.5% [95% CI, 61.9–71.1] in 3-year, and 45.0% [95% CI, 35.9–54.1] *vs*. 56.2% [95% CI, 50.7–61.7] in 5-year; *P* = 0.005, Fig. 3B). After propensity analysis, volatile anesthesia was still associated with both worse overall survival (HR 1.45; 95% CI 1.11–1.89; *P* = 0.006) and recurrence-free survival (HR 1.44; 95% CI 1.11–1.87; *P* = 0.006) (Table 3). After further adjustments with confounders, this relationship between the volatile anesthesia and the overall survival (HR 1.57; 95% CI 1.19–2.07; *P* = 0.001) and recurrence-free survival (HR 1.36; 95% CI 1.04–1.77; *P* = 0.024) was preserved (Table 3). This significant association was consistent with the results of various sensitivity analyses (Supplementary Table S3) and subgroup analyses (Supplementary Fig. S2).

## Discussion

In this retrospective study of 922 patients who underwent elective esophageal surgery for squamous cell carcinoma or adenocarcinoma, we found that intravenous anesthesia with propofol during surgery was associated with both better overall survival and recurrence-free survival rates compared with volatile anesthesia. Furthermore, this relationship remained significant even after adjustment for several potential confounders and was supported by several sensitivity analyses.

Our current findings suggest that the choice of the general anesthesia method could affect long-term outcome after cancer surgery. This result is in accordance with three recent retrospective observational studies that reported a relationship of volatile anesthetics with worse outcome in cancer surgery^11–13^. In one study, propofol anesthesia was reported to increase overall postoperative survival rates compared with sevoflurane in 2,838 patients undergoing surgery for breast, colon, or rectal cancers^11^. In a second study, the relationship of volatile (sevoflurane or isoflurane) *vs*. propofol with long-term survival was evaluated in over 7,000 patients undergoing elective surgery for breast, gastrointestinal tract, gynecology, urology cancer, or sarcoma cancers^12^. After propensity score matching and multivariable analysis, volatile inhalation anesthesia was associated with a HR of 1.46 (95% CI 1.29–1.66) for death compared to intravenous anesthesia with propofol. However, these two studies included various cancer types, but no cancer stages. Indeed, cancer type and stage has been considered an important determinant of survival. Thus, despite their large sample size, the clinical significance of their results may be limited. In a third study, it was reported that, compared to inhalation anesthesia with sevoflurane, intravenous anesthesia with propofol was related to a reduced risk of recurrence during the initial 5 years after surgery in 325 patients undergoing modified radical mastectomy^13^. However, this study may be also limited by small sample size.

In contrast, our study included a more homogeneous population and much larger number of consecutive patients undergoing esophageal cancer surgery. In addition, it included adjusting for a great number of confounders, including perioperative variables known to affect survival such as pathologic stage of cancer, intraoperative transfusions, and postoperative complication, using a comprehensive and accurate data obtained by data abstractors who were blinded to the objectives of this study. Moreover, our findings were consistent using two different statistical risk adjustment methods including multivariable regression and propensity score analysis, and across several sensitivity and subgroup analyses, thus strengthening the validity of our findings.

Although our data cannot explain the underlying mechanism of the beneficial effects of propofol on survival after esophageal cancer surgery, several previous laboratory and animal studies have proposed biological mechanisms for this relationship. Given the possibility of the release of cancer cells into the circulation and potential seeding caused by surgical resection of tumors^5,7^, the immune system, especially cell-mediated immunity, at the time of surgery (i.e. most vulnerable period) may have an important role in postoperative survival and cancer recurrence. It has been reported that volatile anesthetics may suppress the activity of natural killer^14–16^, an important player in the innate immune system and the first-line defense against the invasion of cancer cells, which activity during the perioperative period is inversely related to the development of metastasis^10,17^. Several studies also showed that volatile anesthetics increased the expression of hypoxia-inducible factors and vascular endothelial growth factors that could enhance proliferation, angiogenesis, and metastasis of cancer cells^10,18,19^. In contrast, it has been reported that propofol did not suppress the natural killer activity and reduced the expression of hypoxia-inducible factor^10,15,20^. Moreover, propofol may inhibit matrix metalloproteinases that are the key enzyme involved in breakdown of basement membrane, thus promoting tumor spread^21,22^. Therefore, this preserved immune function related to propofol during surgery could explain the differences in overall and recurrence-free survivals between VA and TIVA groups.

In this study, the incidence of postoperative myocardial dysfunction and sepsis in the VA group was lower than in the TIVA group. This observation is in accordance with recent findings suggesting that volatile anesthetics may provide organ protection by pre- or post-conditioning and anti-inflammatory effects, thus reducing postoperative complications^14,23^. However, these beneficial effects on the short-term outcome could not improve the long-term survival in our study. Thus, because anti-ischemic and anti-inflammatory effects of volatile anesthetics may be protective against organ damage during surgery, but potentially detrimental for cancer patients, the choice of anesthetics may need to be carefully assessed in the overall planning for perioperative care.

Several other perioperative factors including blood transfusion, opioid analgesics, and acute postoperative pain management strategy, which may influence immunomodulation and consequently recurrence or metastases after cancer surgery, may act as confounders. Perioperative allogenic blood transfusion has been reported to have an immunomodulatory effect and hence to be related to an increased risk of cancer recurrence and mortality^10,24,25^. In our study, packed red blood cells given intraoperatively were also associated with both worse overall and recurrence-free survival rates. However, due to our limited data on information related to transfusion (i.e. time of transfusion, use of irradiated or leukocyte-depleted red cell, and postoperative transfusion amounts), further evaluation based on our analysis is not possible. Additionally, opioid administration can suppress both cell-mediated and humoral immunity and promote angiogenesis by direct stimulation of the μ-opioid receptor or activation of vascular endothelial growth factor, which may increase cancer recurrence and mortality^10,26^. Both groups in the present study had received an infusion of the ultrashort-action opioid (remifentanil) during the surgery and rescue opioid analgesics in the postoperative period. However, due to the lack of information on the total amounts of opioids used during the perioperative period in our database, it is impossible to determine their contributory effects in our results. Furthermore, epidural analgesia has been suggested to have a beneficial impact on outcome after cancer surgery by inhibiting the neuroendocrine stress response and reducing perioperative opioid requirements^10,27,28^. In our study, the proportion of patients administered epidural analgesia in the TIVA group was higher than that in the VA group. Thus, although we adjusted for this factor in additional sensitivity analyses, we cannot completely exclude the possibility that this difference of use of epidural analgesia between the two groups could have influenced our results.

Several limitations are inherent in this study. First, because this was a retrospective single-center observational study, our findings could not determine the causal relationship between anesthetics and recurrence and survival after cancer surgery; thus, it should be only deemed as hypothesis-generating. Second, although we performed a multivariable analysis and propensity matching analysis with many variables to obtain reliable results and valuable information, we cannot exclude some unmeasured confounding factors that may be responsible for the result. Third, the management of esophageal cancer patients, including advances in surgical techniques, has evolved over the last few decades, which has resulted in improved outcomes for patients with esophageal cancer. In this study, although the choice of anesthetic agent was made according to the anesthesiologist’s preference, the frequency of use of each agent showed year-to-year variation. Because detailed information about surgical techniques and cancer care advances were not available in our data set, we cannot completely exclude the possibility that advances in cancer care and surgical techniques over the 10-year period of study could influenced the outcomes. Fourth, the cancer subtype included in the present study was mostly squamous cell carcinoma. Esophageal squamous cell carcinoma is the most common subtype in East Asia and has a different outcome from adenocarcinoma^1,2^. Thus, the results might have been different if the subtype was mostly adenocarcinoma. Fifth, because the exact cause-of-death information on our study population was not available, we used all-cause mortality, not cancer-related mortality, as an outcome measure. Thus, these data should be interpreted with caution. Finally, there is significant variability across institutions in the practice of management of patients with cancer^2^. Because this was a single-center study conducted at a tertiary care academic medical center, caution should be taken when generalizing these results to centers with different patient profiles and different practices in the perioperative period.

Despite these limitations, if the relationship between anesthetics and recurrence and survival after cancer surgery is indeed causal, our results may have an important clinical implication for esophageal cancer management. Esophageal cancer is known to be an aggressive malignancy with high recurrence rates after esophagectomy due to the lymphatic and hematogenous metastases facilitated by the abundant lymphatic capillary network in the esophageal mucosa and submucosa^2,8,9^. Furthermore, patients undergoing esophagectomy for cancer are generally exposed to anesthetics for long periods of time due to long operating durations. This suggests that the immunomodulatory effect of anesthetic agents on prognosis in esophageal cancer surgery could be larger than in other cancer surgery. Therefore, in patients undergoing esophagectomy for cancer, intravenous anesthesia with propofol should be preferred over volatile anesthetics as a strategy for improvement in postoperative survival. Given that the poor prognosis of esophageal cancer remained despite improvements in surgical techniques and perioperative management, further prospective randomized studies investigating the survival advantage of intravenous anesthesia with propofol in cancer surgery are urgently required.

## Conclusion

This retrospective observational study of 922 patients who underwent elective esophageal cancer surgery found that intravenous anesthesia with propofol during surgery was related to better postoperative survival rates compared with volatile anesthesia. Further prospective randomized studies are warranted to support our findings.

## Methods

### Study design, participants

This retrospective cohort study was approved by the Institutional Review Board of our institution (AMC IRB 2016–0889). All patients aged 20 years or older who underwent elective esophageal surgery in a tertiary hospital in South Korea between January 2005 and October 2015 were included. Clinical information, including demographic data, comorbidities, laboratory data, medication use, anesthetic management, operative techniques, postoperative management, and morbidity and mortality, were acquired from the Asan Medical Center Esophageal Surgery and Anesthesia Database and from a retrospective review of the computerized patient record system (Asan Medical Center Information System Electronic Medical Record). We excluded patients who (1) had esophagus tumors other than squamous cell carcinoma or adenocarcinoma, (2) underwent other types of surgery simultaneously, and (3) received both forms of anesthesia within the study period. Based on the anesthesia administered during the surgery, patients were divided into either VA group or TIVA group. The present study was performed in accordance with STROBE (Strengthening the Reporting of Observational Studies in Epidemiology) statement^29^. Informed consent was waived by the board due to the retrospective nature of the study without any harm to study subject.

### Anesthesia and postoperative analgesia

The esophageal surgery and perioperative management strategies have been previously described in details^30^. Briefly, according to the preference of the attending anesthesiologists, the patients were given either volatile anesthetic agents (isoflurane, sevoflurane, desflurane) or intravenous anesthetic agent (propofol) during surgery (Supplementary Fig. S1). Opioid (remifentanil) was administered continuously in both groups and the dosage range was adjusted according to the hemodynamic parameters. None of the patients received nitrous oxide. Conventional parameters including heart rate, continuous arterial pressure, central venous pressure, and urine output were used for hemodynamic and fluid management. Packed red blood cells were transfused to maintain a hemoglobin level greater than 8 g/dL (in patients without ischemic disease) or 10 g/dL (in patients with ischemic disease). Postoperative analgesia was achieved using either epidural or intravenous patient-controlled analgesia. Intravenous analgesia was used occasionally if epidural analgesia was inapplicable.

### Surgical resection and pre- and post-operative chemo-radiotherapy

During the study period, patients with resectable esophageal cancer of any stage, except T1/2N0M0, underwent preoperative chemo-radiotherapy. Preoperative chemo-radiotherapy consisted of cisplatin and fluoropyrimidine-based chemotherapy with concurrent radiotherapy at a total dose of 50.4 Gy in 28 fractions of 1.8 Gy each. If hematologic or renal toxicity occurred, chemotherapy and radiotherapy were withheld until evidence of recovery. Subsequent esophagectomy by either Ivor Lewis operation (abdominal-right thoracic approach) or McKeown operation (thoracic-abdominal-cervical approach) with 2-field lymph node dissection was performed within 12 weeks of the conclusion of chemotherapy and radiotherapy.

All operations were performed by experienced surgeons. During the Ivor Lewis operation, the patient was positioned supine. Stomach mobilization and gastric conduit formation with perigastric lymph nodes dissection were carried out. Subsequently, the patient was positioned in the left lateral decubitus position and the intra-thoracic esophagus and para-esophageal lymph glands were removed. The stomach was pulled up into the chest and anastomosis was made between the stomach tube and the remaining esophagus. During the McKeown operation, a right postero-lateral thoracotomy was performed in the left lateral decubitus position. Intra-thoracic esophagus dissection and mobilization were carried out. Subsequently, an incision was made on the left side of the neck and abdomen and the part of the esophagus in the neck, and mobilization of the stomach and gastric conduit formation with perigastric lymph nodes dissection was carried out. The esophagus was divided at the cervical level and the intra-thoracic esophagus and stomach tube were pulled out through the abdomen and chest. Cervical anastomosis was made between the stomach tube and the remaining esophagus.

The stomach was conventionally used as the conduit of choice except in patients with prior gastrectomy, in which case the colon was used instead. The surgeon performed regional dissection of the lymph nodes according to the guidelines for lymph node mapping for esophageal cancer. Antimicrobial agents including cefazolin, isepacin, and metronidazole were administered before surgery. Patients with incomplete surgical resection or lymph node metastasis received postoperative adjuvant chemo-radiotherapy at the physician’s discretion.

### Definitions of variables

The primary outcomes were overall and recurrence-free survivals. Overall survival was calculated from the date of surgery to the date of death from any cause. Recurrence-free survival was calculated from the date of surgery to either the date of first recurrence or the date of death. The last follow-up date was censored from both outcomes. Cancer recurrence was defined by the radiological/histological diagnosis of recurrence after esophagectomy. Data regarding cancer recurrence and death were obtained from outpatient clinics, by a detailed review of all medical records and telephone interviews, or from the National Population Registry of the Korean National Statistical Office. The cut-off date for follow-up was October 31, 2016. Perioperative variables including demographics, comorbidities, laboratory data, medications, intraoperative data, and postoperative data were assessed. The pathologic stage of the esophageal cancer was determined using the TNM classification of the 7^th^ edition of the American Joint Committee on Cancer. Postoperative complications within 90 days after surgery were defined based on the European Perioperative Clinical Outcome definitions or as previously reported^31,32^.

### Statistical Analysis

All data manipulations and statistical analyses were conducted using SAS 9.1 (SAS Institute Inc., Cary, NC, USA) and IBM SPSS Statistics 21.0 (IBM Corp., Armonk, NY, USA). The study sample size corresponded to all patients included in the study and no *a priori* power analysis was conducted. Categorical variables were expressed as numbers and percentages, and continuous variables were expressed as the mean ± standard deviation or median and interquartile range.

In the entire cohort, between-group differences in the perioperative characteristics and postoperative outcomes were compared using the χ^2^ test or Fisher’s exact test for categorical variables and the Student’s t-test or Kruskal-Wallis test for continuous variables whenever appropriate. Univariate and multivariable Cox proportional hazards regression analyses were conducted to assess the independent associations between the anesthetic agents and postoperative overall and recurrence-free survivals. All predictor variables in Table 1 were assessed independently, and variables with a *P* value < 0.20 in the univariate analyses were included in the multivariable analyses. A backward elimination process with a *P* value cut-off of 0.05 was used to develop the final multivariable models.

Moreover, the propensity score matching was performed in order to reduce the effect of a treatment selection bias and potential confounding due to systematic differences between the study groups and to determine the effect of anesthetic agents alone on postoperative overall survival and recurrence-free survival. The propensity scores were estimated without regard to the outcome variables, using a non-parsimonious multivariable logistic regression analysis with the choice of anesthesia as dependent variable and all baseline characteristics shown in Table 1 as covariates. The calipers of width equal to 0.2 of the standard deviation of the logit of the propensity score were used. The discrimination and calibration abilities of each propensity score model were assessed by the C and the Hosmer–Lemeshow statistics. After propensity score matching, we identified two comparable groups of patients (VA: 166 and TIVA: 439, Supplementary Table S1). Matching balance was also assessed with standardized differences for each covariate. In the entire and matched cohorts, multivariable Cox proportional hazards regression analyses were used to assess the hazard ratios (HRs) with 95% confidence interval (CI) of the relationships between the anesthetic agents and postoperative overall and recurrence-free survivals.

Overall and recurrence-free survival rates in the entire and matched cohorts were compared between the study groups using the Kaplan-Meier curves with log-rank test. Additionally, several sensitivity and subgroup analyses were performed to assess the robustness of the findings. All the reported *P* values were 2-sided, and *P* values < 0.05 were considered statistically significant.

## Electronic supplementary material

Supplementary file
