# Supplementary material for: Impact of anesthetic agents on overall and recurrence-free survival in patients undergoing esophageal cancer surgery: A retrospective observational study
Source: Sci Rep. 2017 Oct 25;7:14020. doi: 10.1038/s41598-017-14147-9 (PMC5656640; doi:10.1038/s41598-017-14147-9)

-Supplementary Materials-

Impact of anesthetic agents on overall and recurrence-free survival in patients undergoing esophageal cancer surgery: A retrospective observational study

In-Jung Jun, Jun-Young Jo, Jong-Il Kim, Ji-Hyun Chin, Wook-Jong Kim,

Hyeong Ryul Kim, Eun-Ho Lee, In-Cheol Choi

Table of Contents

Table S1: Baseline characteristics in matched patients after propensity scoring

Table S2: Variables associated with overall survival after esophageal cancer surgery in Cox proportional hazard regression model

Table S3: Comparisions of results for primary analysis of overall survival and recurrence-free survival compared with sensitivity analyses

Figure S1: Use of anesthetic techniques according to year of surgery

Figure S2: Association between anesthesia type and overall survival (A) and recurrence-free survival (B) in subgroups

Table S1. Baseline characteristics in matched patients after propensity scoring

Variable	VA group	TIVA group	STD
N	166	439	
Demographics			
Age (yr)	62.9 ± 7.1	62.8 ± 7.2	0.016
Male gender	159 (95.8)	411 (93.6)	0.097
Clinical characteristics			
Body mass index (kg/m^2)	23.0 ± 3.0	23.1 ± 2.9	0.020
ASA class			0.085
I	15 (9.0)	33 (7.5)	
II	149 (89.8)	397 (90.4)	
III	2 (1.2)	9 (2.1)	
Medical history			
Diabetes mellitus	21 (12.7)	66 (15.0)	0.069
Hypertension	59 (35.5)	156 (35.5)	0.0001
Dyslipidemia	13 (7.8)	37 (8.4)	0.022
Coronary artery disease	2 (1.2)	6 (1.4)	0.014
Cerebral vascular disease	5 (3.0)	18 (4.1)	0.059
Peripheral vascular disease	2 (1.2)	11 (2.5)	0.097
COPD	3 (1.8)	8 (1.8)	0.001
eGFR < 60 mL/min/1.73 m^2	7 (4.2)	20 (4.6)	0.017
Liver disease	13 (7.8)	39 (8.9)	0.038
Smoker, current	41 (24.7)	114 (26.0)	0.029
Alcohol status	145 (87.4)	380 (86.6)	0.023

Chemo-radiation therapy	69 (41.6)	178 (40.6)	0.021
Laboratory data			
Hematocrit (%)	37.7 ± 4.9	37.8 ± 5.0	0.021
Creatinine (mg/dL)	0.9 [0.8–1.0]	0.8 [0.7–0.9]	0.049
Total bilirubin (mg/dL)	0.5 [0.4–0.7]	0.6 [0.4–0.7]	0.075
Albumin (g/dL)	3.7 ± 0.4	3.7 ± 0.4	0.027
FEV_1 / FVC	72.2 ± 9.9	72.5 ± 9.3	0.028
Medication			
ACEI or ARB	27 (16.3)	73 (16.6)	0.010
Beta blocker	10 (6.0)	32 (7.3)	0.051
Calcium channel blocker	28 (16.9)	84 (19.1)	0.059
Insulin	21 (12.7)	63 (14.4)	0.050
Oral hypoglycemic agent	16 (9.6)	45 (10.3)	0.021
Statin	12 (7.2)	37 (8.4)	0.045
Aspirin	10 (6.0)	31 (7.1)	0.042
Plavix	2 (1.2)	10 (2.3)	0.082
Diuretics	9 (5.4)	28 (16.4)	0.041

Data are expressed as number of patients (%), mean ± standard deviation, or median [first-third quartiles].

VA = volatile inhalational anesthesia; TIVA = total intravenous anesthesia; STD = standardized difference; ASA = American Society of Anesthesiology; COPD = chronic obstructive pulmonary disease; eGFR = estimated glomerular filtration rate; FEV_1 = forced expiratory volume in 1 second; FVC = forced vital capacity; ACEI = angiotensin-converting enzyme inhibitor; ARB = angiotensin receptor blocker.

Table S2. Variables associated with overall survival after esophageal cancer surgery in Cox proportional hazard regression model

Variable	Hazard Ratio	95% CI	*P* value
VA group	1.58	1.24–2.01	< 0.001
Age (yr)	1.02	1.00–1.03	0.011
Body mass index (kg/m^2)	0.91	0.88–0.95	< 0.001
Diabetes mellitus	1.29	0.99–1.67	0.059
Preoperative chemo-radiation therapy	1.61	1.26–2.06	< 0.001
Preoperative serum albumin level (g/dL)	0.53	0.40–0.70	< 0.001
Pathologic stage of cancer			
I	1.36	0.96–1.92	0.084
II	2.85	2.09–3.88	< 0.001
III	6.31	4.51–8.81	< 0.001
IV	8.70	4.41–17.16	< 0.001
pRBC (unit) given intraoperatively	1.10	1.02–1.20	0.016
Postoperative myocardial dysfunction	1.37	1.06–1.78	0.015
Postoperative sepsis	1.59	1.20–2.11	0.001

CI = confidence interval; VA = volatile inhalational anesthesia; pRBC = packed red blood cell.

Table S3. Comparisions of results for primary analysis of overall survival and recurrence-free survival compared with sensitivity analyses

	VA group	
	Adjusted Hazard Ratio (95% CI)	*P* value
Overall survival		
Primary model*	1.58 (1.24–2.01)	< 0.001
Adjusted for epidural analgesia	1.55 (1.22–1.98)	< 0.001
Adjusted for one lung ventilation time	1.59 (1.26–2.04)	< 0.001
Adjusted for year of surgery	1.61 (1.26–2.06)	< 0.001
Adjusted for type of surgery	1.57 (1.24–1.99)	< 0.001
Adjusted for minimally invasive surgery	1.58 (1.24–2.00)	< 0.001
Adjusted for type of esophageal carcinoma	1.58 (1.24–2.01)	< 0.001
Pre- and intra-operative variables only	1.49 (1.18–1.89)	0.001
Recurrence-free survival		
Primary model†	1.42 (1.12–1.79)	0.003
Adjusted for epidural analgesia	1.40 (1.10–1.77)	0.005
Adjusted for one lung ventilation time	1.43 (1.13–1.80)	0.003
Adjusted for year of surgery	1.42 (1.12–1.79)	0.004
Adjusted for type of surgery	1.42 (1.12–1.79)	0.003
Adjusted for minimally invasive surgery	1.41 (1.12–1.79)	0.004
Adjusted for type of esophageal carcinoma	1.41 (1.12–1.78)	0.004
Adjusted for postoperative CRT	1.42 (1.12–1.79)	0.003

*: adjusted by age, body mass index, diabetes mellitus, preoperative chemo-radiation therapy, preoperative albumin levels, pathologic stage of cancer, pRBC given intraoperatively, postoperative myocardial dysfunction (except pre- and intra-operative variable model), and

postoperative sepsis (except pre- and intra-operative variable model).

†: adjusted by age, body mass index, preoperative chemo-radiation therapy, preoperative albumin levels, pathologic stage of cancer, and pRBC given intraoperatively.

VA = volatile inhalational anesthesia; CI = confidence interval; CRT = chemo-radiation therapy; pRBC = packed red blood cell.

Figure S1: Use of anesthetic techniques according to year of surgery

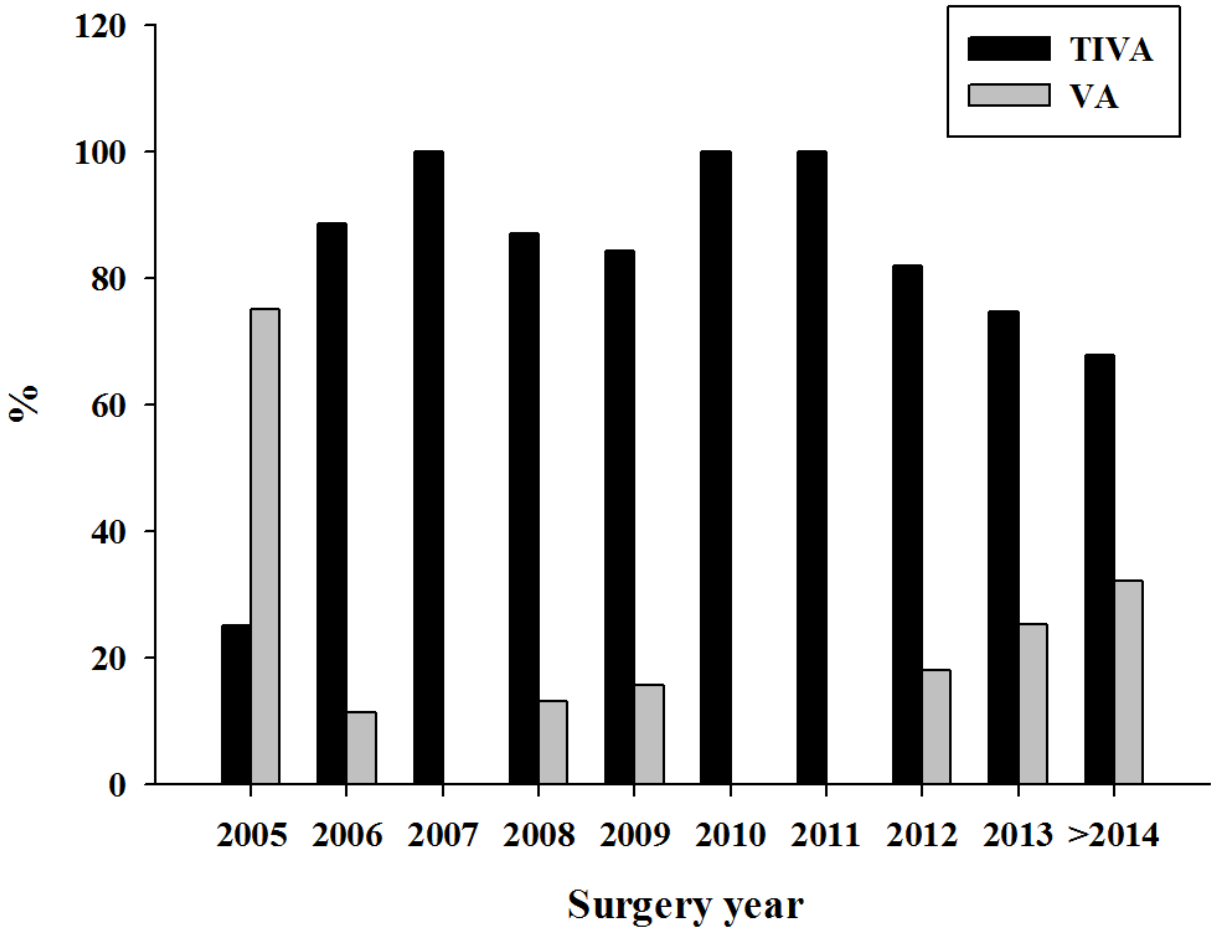

Number of cases	64	53	51	69	70	80	89	133	111	202
TIVA	16	47	51	60	59	80	89	109	83	137
VA	48	6	0	9	11	0	0	24	28	65

Figure S2: Association between anesthesia type and overall survival (A) and recurrence-free survival (B) in subgroups

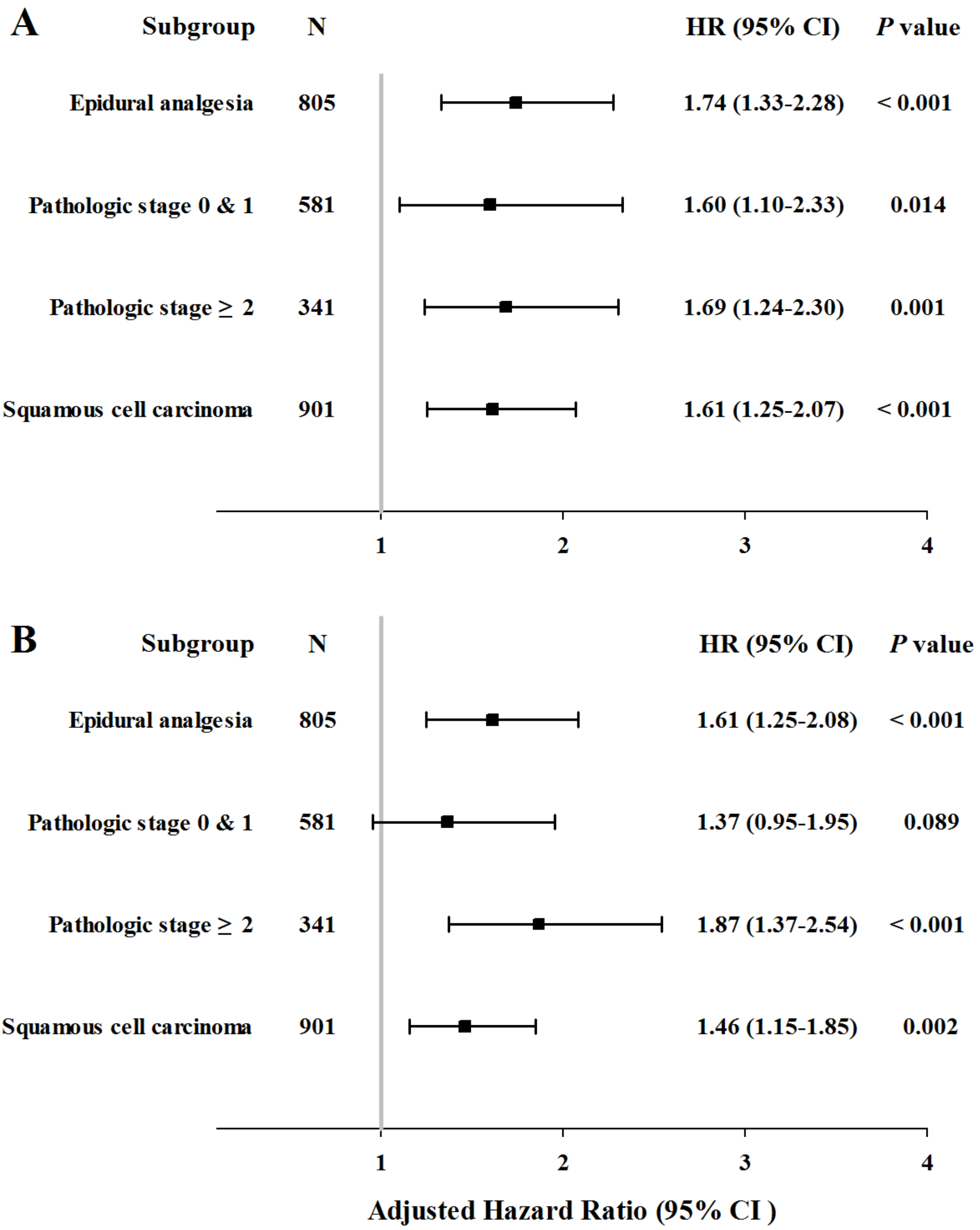

Supplement: Supplementary file 1 — Supplementary file [file 41598_2017_14147_MOESM1_ESM.pdf]
